# Driver state detection and recognition in conditional automated driving: A review

**DOI:** 10.1371/journal.pone.0358700

**Published:** 2026-09-17

**Authors:** Haoran Wu, Timothy Tettey Nartey, Guangjun Wan, Yugang Wang, Linli Xu, Xun Zhou

**Affiliations:** 1 College of Automotive Engineering, Hubei University of Automotive Technology, Shiyan, China; 2 Key Laboratory of Automotive Power Train and Electronic Control, Hubei University of Automotive Technology, Shiyan, China; 3 Department of Breast and thyroid Surgery, Sinopharm Dongfeng General Hospital, Hubei University of Medicine, Shiyan, China; Yanshan University, CHINA

## Abstract

SAE Level 3 automated driving systems assume full control of the dynamic driving task within their operational design domain. Ensuring a safe and timely transition from automated to manual driving therefore necessitates continuous monitoring and assessment of driver state. This review presents a synthesis of 85 studies published mainly between 2021 and 2025. A PRISMA 2020-guided search was conducted in ScienceDirect, Web of Science, SpringerLink and IEEE Xplore, with the final search completed on 15^th^ September 2025. Only English-language publications that examined driver-state detection, recognition or assessment in driving scenarios relevant to conditional automation were considered. Studies published before 2021 were excluded, unless they provided foundational contributions. This review was not registered. This review aims to (1) identify driver-state constructs that have been investigated in conditional automated driving, examining how they are defined and operationalized; (2) characterize sensing modalities used for driver-state monitoring; (3) evaluate computational approaches including multimodal fusion used in previous studies; (4) examine performance metrics, validation strategies and real-time feasibility; (5) identify methodological limitations, reporting inconsistencies and priorities for future development and deployment. This study suggests the standardisation of Level 3 datasets. It also calls for harmonised operational definitions and more representative sampling. It finally advises the performance of more longitudinal on-road validation. This work was supported by the National Natural Science Foundation of China.

## 1 Introduction

Automation of consumer automobiles over the past few years has been an accelerated process. This has evolved to different levels of shared control, a process which started at the manual level. The Society of Automotive Engineers (SAE) taxonomy labels Level 0 as no driving automation (the manual level) and Level 5 as full automation [1]. Level 3, also known as conditionally automated vehicles, allows the driver to surrender the driving task to the vehicle system. The driver must however resume control at the time when the system transmits a takeover request [2]. The preparedness of the driver during these transitions is a fundamental factor determining whether the transitions will be safe.

There are several factors that affect the quality of driver takeover. These include driver distraction [3], drowsiness [4], fatigue [5,6], emotions such as anger [7], frustration [8,9] among others. Distraction disrupts both visual processing and cognitive processing [3]. Cognitive load is defined as the quantity of information the working memory is required to store and operate on at a given time [10]. An unreasonable mental workload hinders the ability of the driver to respond effectively during the state of overload [11,12]. Takeover requests may cause increased mental load, increasing the risk of mistakes [13]. The urgency of auditory warnings as well as visual load have a negative impact on takeover performance. especially when drivers have to deal with excessive visual information or low-visibility conditions [11,14–17].

Takeover behavior has also been linked to personality traits [18]. Driving stability depends on emotion regulation, and willingness to takeover is dependent on trust in automation [19]. Outcomes are dependent on the understanding of the limitations of automation by the driver; increased knowledge will lead to more anticipation of the potential failure of automation and introduce safer transition [20]. When faced with an emergency, it is necessary to establish the decision as to whether the system or the driver should take precedence. This would be possible through a clear perception of the driver awareness [21].

Detection of driver’s awareness, henceforth referred to as Driver-state detection, is important in the avoidance of road crashes [22]. In detecting driver state, timing is an important factor to consider. It was found that with non-driving related tasks (NDRTs), the average time needed to regain control of a vehicle is about six seconds [10]. Within this period, the consciousness of the driver should be assessed to avoid collision.

The main categories of sensing modalities that help to evaluate driver state are vision-based modalities, physiological-based modalities and vehicle-performance modalities. Vision-based approaches are based on the behavioral measures, including eyelid closures, the number of blinks, expressions, and head movements [23]. Physiological sensing modalities comprises detection of internal states through electroencephalography (EEG), electrooculogram (EOG), electrocardiogram (ECG), electromyography (EMG), respiration and body temperature [24,25]. Vehicle-performance modalities utilize such variables as steering angle, velocity, and standard deviation of lateral position (SDLP). A combination of sensing modalities has exhibited increased strength and accuracy [26]. Subjective behavioral instruments, such as the F‑DBQ [27], extend measurement scope by examining psychological factors related to accidents, including fatigue and stress.

Varied collection of computational procedures is utilized to classify or evaluate driver condition. Traditional machine-learning models such as neural network, random forests [12], and a support-vector-machine have been used alongside deep-learning models, like hyperbolic-tangent long short-term memory (LSTM) hybrids [4] and Custom Visual Geometry Group-19 (CVGG-19) architecture [28]. Fragmentation of the literature in this area has been brought about by the heterogeneity of sensing technologies and methods of analysis, within such subject areas as psychology, human factors and computational modeling. This disintegration makes it difficult to come up with a uniform standard of evaluating driver state for takeover preparedness in Level 3 vehicles.

Several reviews have examined aspects of driver monitoring and automated driving, but none provides the integrated, PRISMA-guided synthesis offered by the present work. Dong et al. [29] and Jo et al. [30] established foundational frameworks for driver inattention monitoring and drowsiness detection, respectively, but predate the widespread adoption of Level 3 automation and do not address the takeover-specific context that defines the safety challenge at SAE Level 3. More recent reviews have examined individual modalities – for example, physiological sensing for fatigue detection [25] – but have not systematically compared vision-based, physiological, vehicle-performance, and multimodal fusion approaches within a unified methodological framework. Furthermore, existing reviews have not applied formal risk-of-bias assessment tools (The Quality Assessment of Diagnostic Accuracy Studies 2 (QUADAS-2), Risk-of-Bias Visualization (RoBVIS)) to driver-state detection research, nor have they systematically evaluated the metric reporting practices – accuracy, F1-score (the harmonic mean of precision and recall), Area Under the Curve (AUC), sensitivity – that determine whether performance claims are meaningful for safety-critical deployment. This review addresses these gaps by: (1) providing PRISMA 2020-compliant synthesis of driver-state detection related to SAE Level 3 conditional automation; (2) comparing all three major sensing modalities, together with their fusion within a single analytical framework; (3) applying formal bias assessment to evaluate methodological quality across 85 studies; and (4) identifying the metric reporting inconsistencies that currently prevent cross-study comparison and benchmarking.

This review is specifically scoped to SAE Level 3 conditional automation and the driver-state conditions relevant to takeover readiness. It does not cover driver monitoring in fully autonomous (Level 4–5) systems, driver assistance systems (Level 1–2), or driving under non-automated conditions, except where such studies provide validated sensing or algorithmic methods directly applicable to the Level 3 context.

The review is structured as follows: Section 2 outlines the methodology while section 3 summarizes the current research landscape. Section 4 discusses the main findings. Section 5 outlines future research directions and research gaps and section 6 concludes the review.

## 2 Methodology

A comprehensive literature search using four electronic databases, ScienceDirect, Web of Science, SpringerLink and IEEE Xplore, was performed. The final search was completed on 15^th^ September 2025. Papers published within the years 2021–2025 were examined to investigate driver-state detection methods and strategies. The following disciplines was the scope of the study; engineering (with more emphasis on electrical engineering and computer engineering), computer science and transportation studies.

The search queries were developed taking into account phrases found in titles, abstracts, author keywords, and keywords plus in order to increase query relevancy. Queries included terms to identify the intended system, referencing “driver state detection,” “driver monitoring,” “takeover readiness,” “conditional automated driving,” and “Level 3 automation,” among other relevant expressions. Terms associated with the constructs of interest were included to further define the scope, such as “fatigue,” “drowsiness,” “distraction,” “trust,” “workload,” and “takeover performance,” as well as terms associated with the methods and signals used, such as “EEG,” “electrooculography,” “heart rate variability,” “steering entropy,” and “eye tracking”. Table 1 shows the final queries, adapted across all four databases

**Table 1:** Keyword block used across all four databases.

• (driver state OR driver condition OR driver status OR distraction OR drowsiness) AND • (Level 3 OR SAE Level 3 OR conditional automation) AND • (recognition OR detection OR classification)

### 2.1 Eligibility criteria

In this review, papers that satisfied the following criteria were considered;

1. Papers that examined driver-state detection, recognition or assessment in driving scenarios
2. Papers that used sensing methods like physiological or behavioral assessments, camera-based systems, vehicle dynamic sensors or multi-sensor fusion techniques
3. Papers that used validated behavioral instruments and offered evidence in line with driver-state detection for takeover preparedness
4. Papers that reported empirical behavioral or psychometric outcomes, provided they gave quantitative results or classification measures. Algorithmic frameworks for real-time inference were also considered

Studies were excluded from this review if they were 1) Published before 2021, unless identified as foundational contributions to driver-state monitoring frameworks (two such exceptions, [29,30] were retained: [29] was retained due to its provision of a broad system-level review of driver inattention for intelligent vehicles, classifying inattention and organizing monitoring approaches, supporting the sensing-modality structure used in this review; [30] was retained for providing early empirical evidence of driver-state detection using feature-level fusion and user-specific classification; 2) Not published in English; 3) Studies focused exclusively on fully autonomous (SAE Level 4–5) or fully manual (Level 0–1) driving, with no relevance to conditional automation or takeover transitions.

### 2.2 Selection process

**Fig 1:**
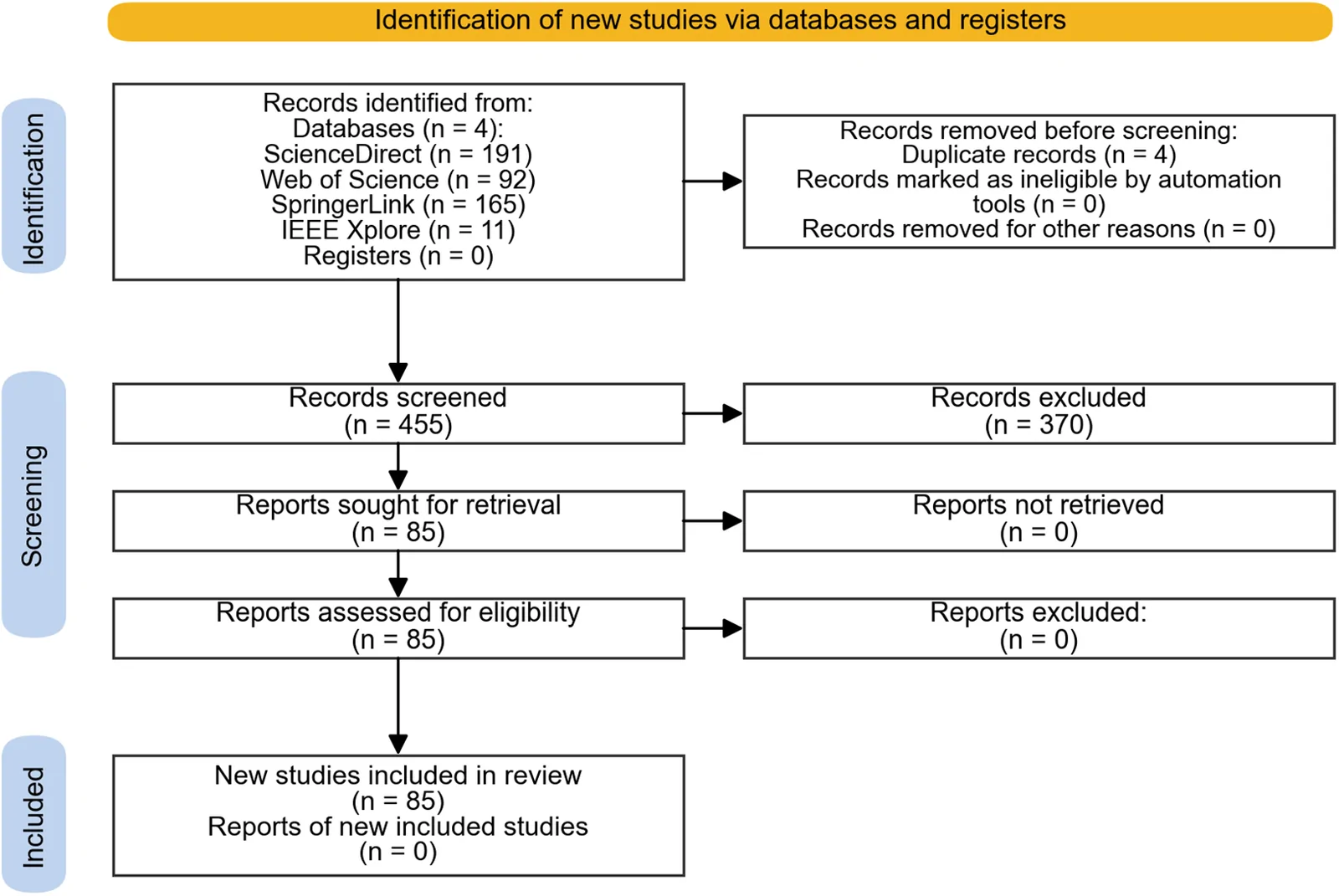
PRISMA flow diagram for identified studies.

An initial collection of 459 records was obtained by exporting and consolidating the identified records from each database in EndNote 21. All retrieved records were exported into EndNote 21 for reference management. Duplicate records were identified and removed using EndNote’s automated duplicate detection function, followed by manual verification to ensure that records representing the same study were not retained multiple times. Four duplicates were thus removed by deduplication, leaving 455 unique records. Following duplicate removal, titles and abstracts were screened against predefined eligibility criteria by the review team. Screening decisions were subsequently checked within a team of four reviewers to ensure consistency with the inclusion and exclusion criteria. Records considered potentially relevant were retrieved for full-text assessment. Full-text articles were evaluated based on their relevance to driver-state detection or assessment in conditional automated driving, the sensing or assessment modality used, and the reported behavioral, physiological, psychometric, computational, or takeover-related outcomes. During title and abstract screening, records were excluded when they did not align with the predefined scope of this review. The main reasons for exclusion included studies unrelated to driver-state detection or assessment; studies focusing on general driving behavior without relevance to takeover preparedness; studies investigating fully autonomous (SAE Level 4–5) or fully manual (SAE Level 0–1) driving without transferable relevance to conditional automation. Any uncertainties or disagreements regarding study eligibility were discussed among the reviewers and resolved through consensus. No quantitative measures of inter-rater agreement were used. The selection procedure is summarized in the PRISMA flow diagram (Fig 1). The data items of interest extracted from the papers are also listed in Table 2.

**Table 2:** List of variables considered for data extraction.

Variables
Classification accuracy Sensitivity F1-score AUC Reaction-time impacts Sensor type Algorithmic approach Dataset origin Participant demographics Sample sizes

### 2.3 Risk-of-bias assessment

Although driver-state detection is primarily an automotive human-factors research area, many of its assessment approaches rely on physiological and behavioral measurements that share characteristics with biomedical diagnostic evaluations. Signals such as EEG, ECG, EOG, heart-rate variability (HRV) and ocular behavioral measures are used to infer latent human states. QUADAS-2 was thus considered an appropriate methodological foundation because it provides a structured framework for evaluating studies involving an index test, reference standard, and outcome interpretation. However, the original framework was adapted to reflect the specific requirements of driver-state detection research. The domains assessed included driver selection, index test, reference standard and flow and timing, as shown in Table 3. Ratings were categorized as low, high or moderate. QUADAS-2 ratings were summarized using RoBVIS.

**Table 3:** Risk of bias domains and their definitions.

Domains	Definition
D1	Description of driver selection
D2	Description of the index test, how it was conducted and interpreted
D3	Description of the reference standard, how it was conducted and interpreted
D4	Description of any driver(s) who did not receive the index test(s)

Qualities that characterise a low risk of bias for all domains are also summarised in Table 4.

**Table 4:** Qualities that characterize low-risk of bias.

Domains	Low-risk characteristics
Domain 1	Drivers were randomly selected or consecutively selected Inclusion/exclusion criteria were clearly reported Avoidance of a case-control or convenience sampling Inclusion of all relevant subgroups (age, experience levels)
Domain 2	The index test was used uniformly for all drivers Pre-specification of the thresholds of the test Standardization of test conditions
Domain 3	Using reference standards that are widely accepted and valid Using the same reference standard for all drivers
Domain 4	Administering the same index test and reference standard Short or no time interval between the index test and reference standard Analyzing data consistently for all drivers Missing data were minimal or properly addressed

### 2.4 Synthesis method

A quantitative meta-analysis was not conducted due to substantial heterogeneity in study designs, driver-state definitions, experimental contexts. However, findings were synthesized narratively and thematically. Studies were grouped by their experimental contexts and synthesized according to sensing modalities, algorithmic approaches, reported metrics and relevance to takeover preparedness. Table and visual summaries were used to organize the information and present patterns across study characteristics.

## 3 Current research landscape

**Fig 2:**
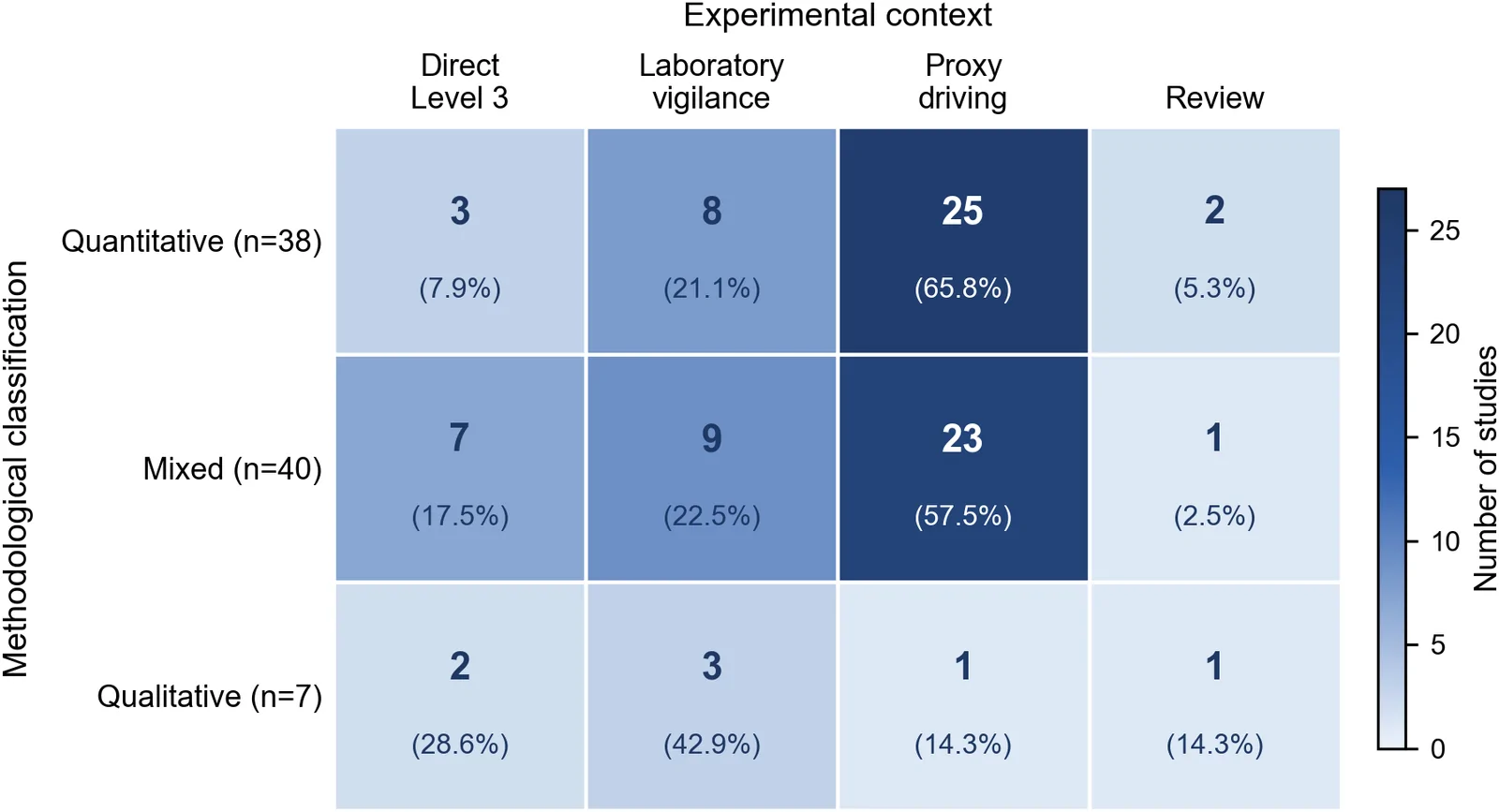
Distribution of studies by methodology and experimental context.

**Fig 3:**
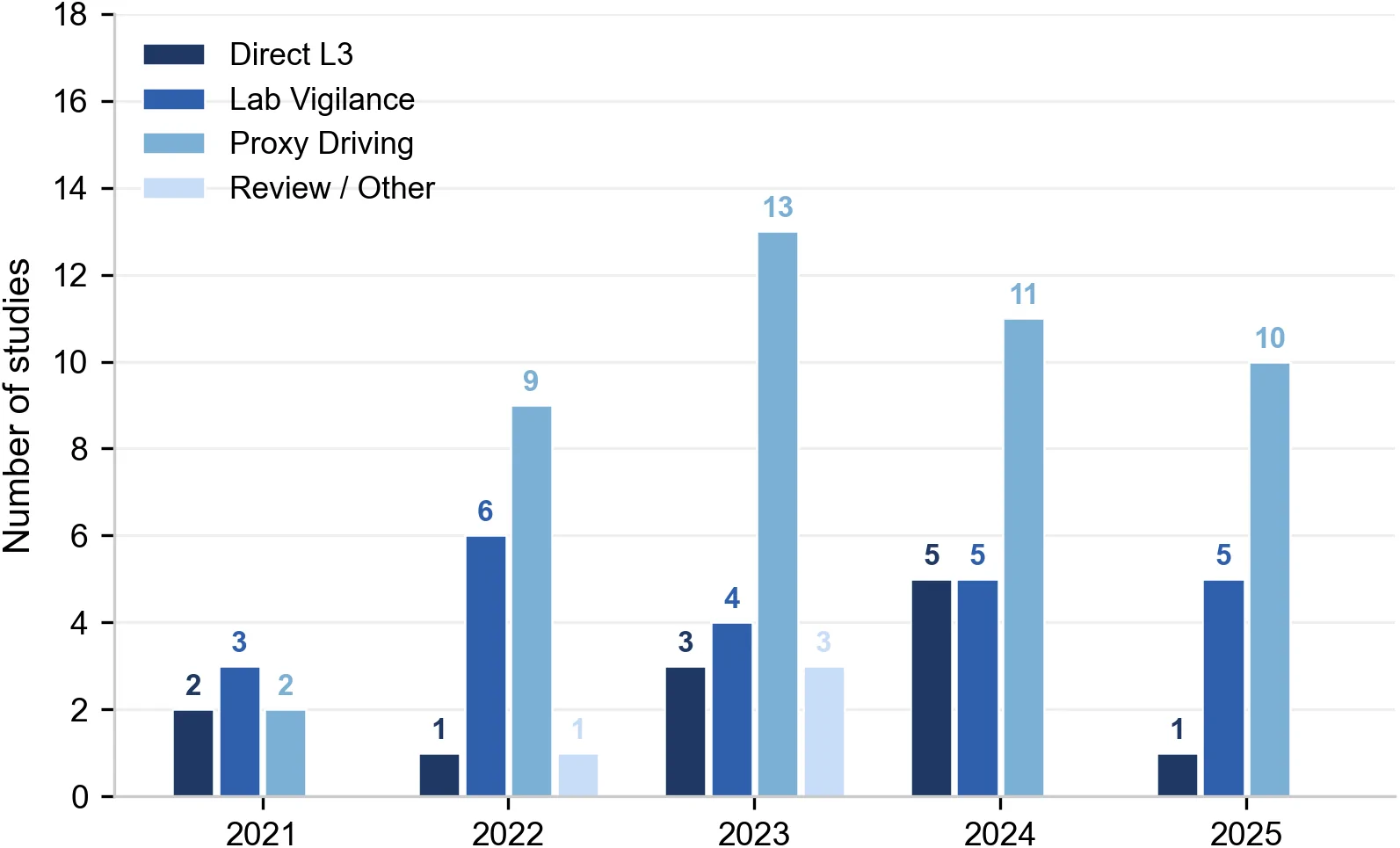
Annual distribution of included studies by experimental context.

In all literature obtained, investigations were divided into 4 large categories: direct Level 3 automation, laboratory-vigilance, proxy-driving and review. Direct Level 3 automation papers had a specific situation that involved SAE Level 3 automation and 12 papers of the total 85 papers were categorized in this. Laboratory-vigilance projects conducted controlled experiments under simulators or laboratory condition and applied validated tools to assess the driver states by behavioral and physiological reactions and 20 papers came under this category. The proxy-driving studies entailed the participants to undertake procedures that are known to be approximating the attentional requirements, awareness rate, or load on the visions or perceptions as external machines assessed the driver-state attention and mental load; a total of 49 papers were included here. 4 papers were categorized under review (Fig 2). The annual distribution of the studies across these experimental contexts is also shown in Fig 3.

**Fig 4:**
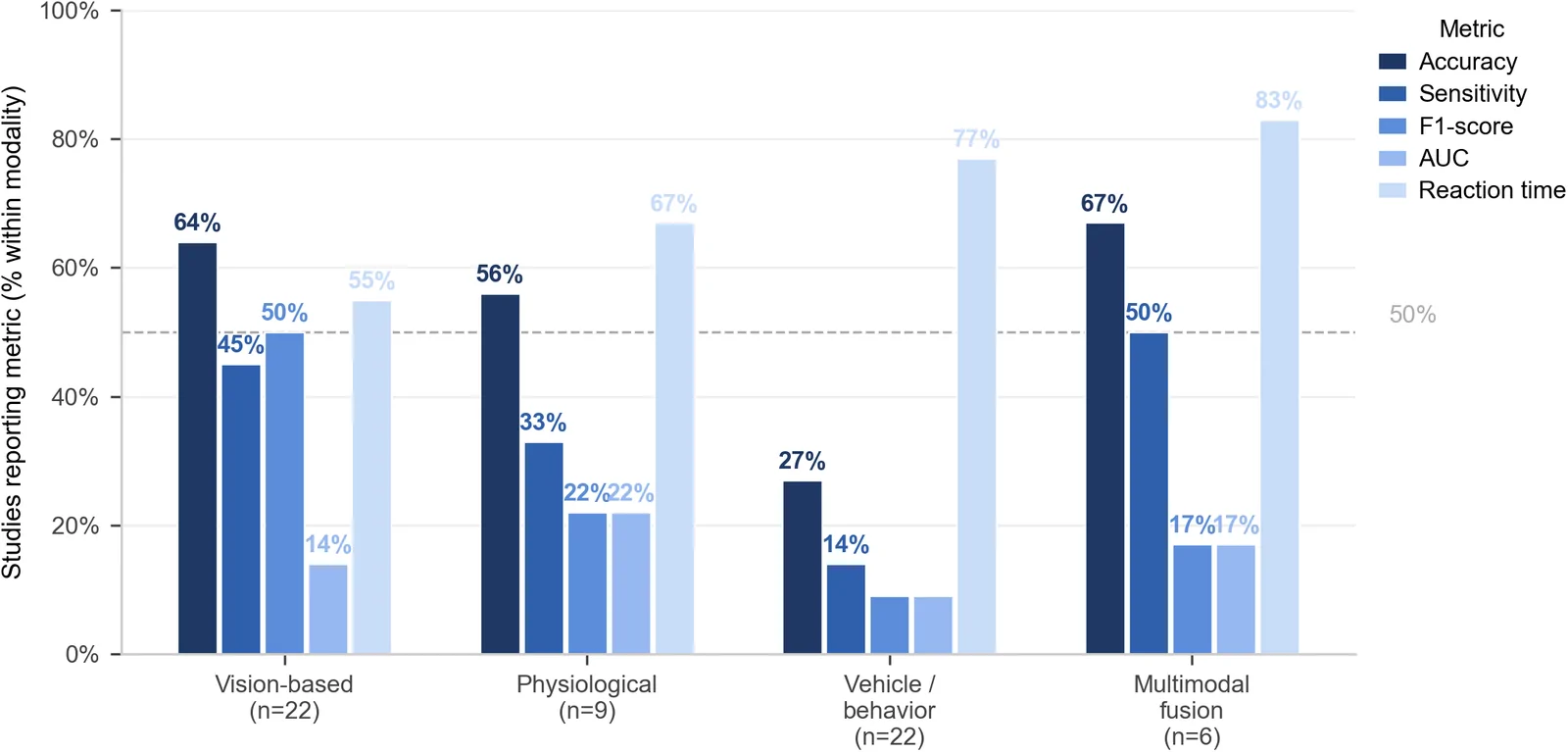
Performance metric reporting rates by sensing modality.

**Fig 5:**
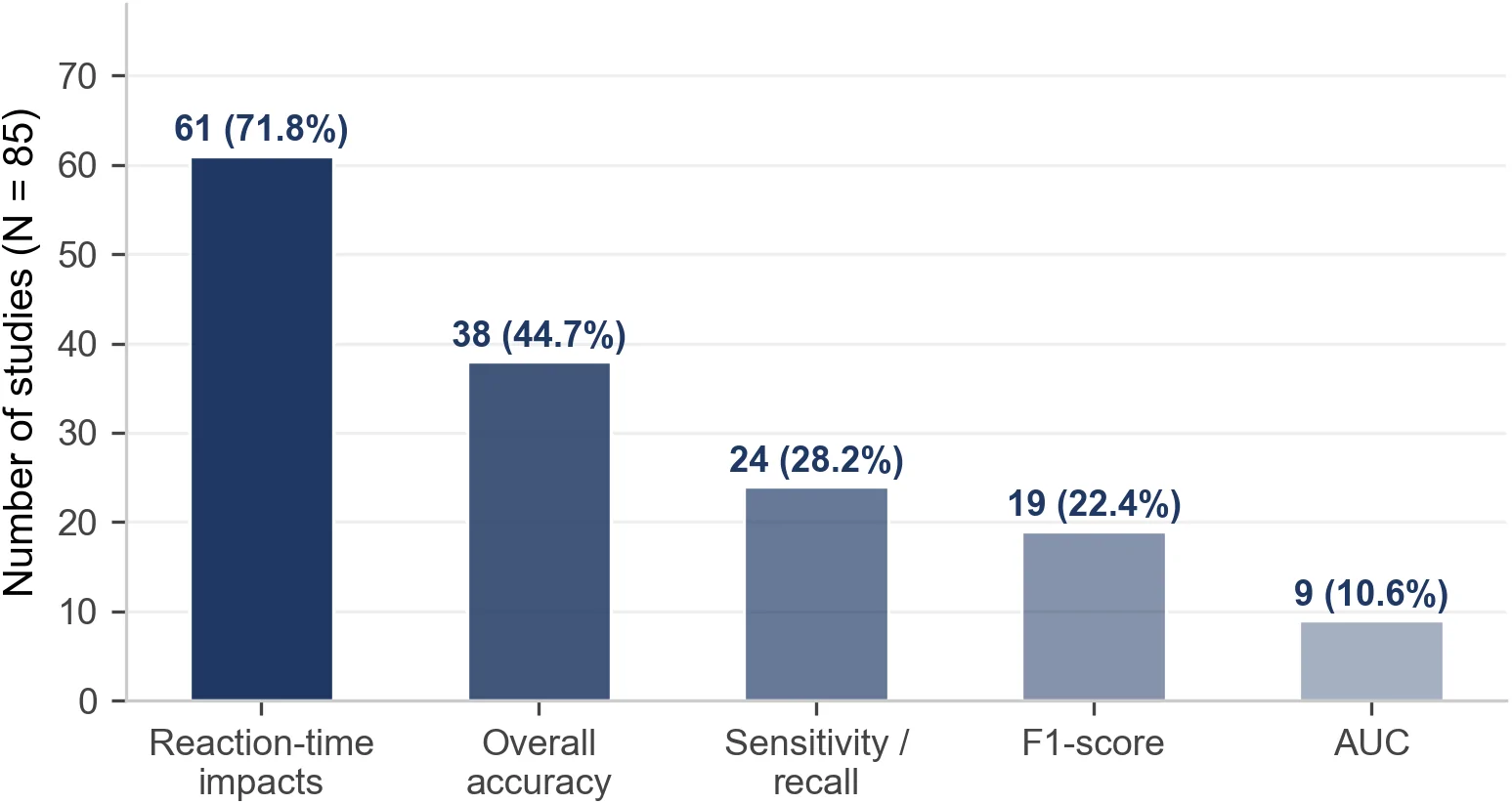
Performance metrics reported across reviewed studies.

The performance of sensing modalities reported was evaluated using one of the following metrics; 1) Accuracy 2) Sensitivity 3) F1 Score 4) AUC or 5) Reaction-time. Figs 4 and 5 respectively illustrate the distribution of the performance metrics across studies reviewed and the relationship between sensing modalities and the performance metrics

Across the 85 studies, several distinct algorithmic families were identified. Deep learning architectures – including Convolutional Neural Networks (CNNs), 3D-CNNs, Inception-Residual Network (ResNet) variants, attention-based networks, YOLO variants, and hybrid CNN-LSTM models – were present in distraction, emotion, and drowsiness detection using image and video data. Physiological deep models applied stacked autoencoders, LSTM, and Bi-LSTM to EEG, ECG, EDA, and HRV signals for fatigue, vigilance, and mental workload classification. Classical machine learning methods – including SVM, Random Forest, KNN, XGBoost, LightGBM, AdaBoost, PCA + SVM, RBF-SVM, and clustering algorithms (GMM, FCM) – were adopted for physiology-based detection and vehicle-performance tasks. Multimodal fusion models applied feature-level or decision-level fusion combining visual, physiological, radar, vehicle, and environmental inputs, and are increasingly applied to Level 3 driver-monitoring challenges. Statistical and behavioral modeling approaches – including ANOVA, GLMM, mediation models, polynomial regression, entropy measures, and SAGAT-based situation-awareness analysis, without machine learning – focused on behavior, attention, workload, and takeover dynamics. Psychometric and non-algorithmic approaches using questionnaire-based modeling (SEM, CFA, regression, correlations) without sensing or classification were applied in a further group of studies.

Table 5 summarizes the major vision-based deep learning methods used in driver-state detection studies. These include CNN-based studies, spatiotemporal models and attention-enhanced networks. The table highlights the dominance of convolutional architecture in extracting facial, behavioral and environmental features from image and video data.

**Table 5:** Vision-based deep learning methods.

Method	Description	Reference(s)
Custom CNN / VGG-style CNN	Customized VGG-19 or related CNN backbones for facial or driver-state classification.	[23,28,31,32]
YOLO-based detection	YOLO-family models used for object, vehicle, or road-feature detection within driver-state or driving-scene pipelines.	[12,33,34]
3D-CNN + LSTM	Spatiotemporal video modeling for fatigue, yawn, or drowsiness behavior recognition.	[35]
Inception-ResNet variants	Refined Inception-ResNet A/B-style architecture for face-based recognition.	[36]
Attention-based CNNs	CNNs enhanced with attention modules or multi-head attention.	[37,38]
CNN-BiLSTM / CNN-LSTM hybrids	Hybrid convolutional-recurrent architectures for distracted or drowsy-driving recognition.	[39–41]
Other vision DL backbones	DeepLabv3 + , UNet, SSD MobileNetV2, and VGG16 used in multi-feature roadway or vehicle-perception pipelines.	[34]

*CNN- Convoluted Neural Network, VGG- Visual Geometry Group, YOLO- You Only Look Once, LSTM- Long Short-Term Memory, Bi-LSTM- Bidirectional Long Short-Term Memory, DL- Deep Learning

Table 6 presents deep learning methods which were applied to physiological signal analysis. These include LSTM-based temporal models and multi-branch convolutional architecture for biosignal fusion. These methods emphasize the importance of temporal dynamics in interpreting EEG, ECG and EDA signals.

**Table 6:** Physiological deep learning methods.

Method	Description	References
Stacked autoencoder + attention-LSTM	Deep physiological sequence model for drowsiness or vigilance classification.	[4]
LSTM	Biosignal temporal modeling using LSTM layers.	[24]
Deep CNN for biosignal fusion	Multi-branch deep CNN on EEG, EDA, and ECG with feature-level fusion.	[26]
MLP / feed-forward neural network	Neural-network modeling on naturalistic multi-sensor driving streams.	[42]
LSTM on naturalistic monitoring data	Temporal modeling of driver risk or state from longitudinal driving streams.	[42]

*MLP- Multilayer Perception.

Table 7 outlines classical machine learning techniques used across the reviewed studies, including SVM, Random Forest and clustering algorithms. The advent of deep learning methods does not render classical machine learning methods redundant. This is due to their ease of interpretability, lower computational cost and effectiveness on structured physiological and vehicle data.

**Table 7:** Classical machine learning methods.

Method	Description	References
SVM	Used in physiology-based and multimodal driver-state classification.	[12,30,43–46]
RBF-SVM / RBF-SVR	Kernel SVM or support-vector regression in drowsiness or multimodal prediction.	[30,46,47]
Random Forest (RF)	Classical ensemble classifier in physiological or multimodal driver monitoring.	[12,21,43,48]
KNN	Shallow classifier for fatigue, performance, or RC-car proxy tasks.	[43,45,48]
XGBoost	Gradient-boosted tree model in physiological fatigue detection.	[43]
LGBM	Gradient boosting for safety or fatigue prediction.	[21,43,49]
AdaBoost	Used in hybrid vision or risk pipelines.	[21,50]
PCA + SVM	Feature reduction plus SVM verification or workload classification.	[44]
Clustering: k-means	Unsupervised grouping or thresholding before classification or interpretation.	[50–53]
Clustering: FCM	Used for workload- or trust-related grouping.	[44,49]
Clustering: GMM	Unsupervised clustering tied to gaze modeling.	[32]
Other shallow ensembles	Decision Tree, Gradient Boosting, Naive Bayes, and CatBoost used as comparators or ensemble members.	[21,47,48]

*SVM- Support Vector Machine, RBF- Radial Basis Function, KNN- K-Nearest Neighbor, XGBoost- Extreme Gradient Boosting, LGBM- Light Gradient Boosting Machine, PCA- Principal Component Analysis, FCM- Fuzzy C-Means, GMM- Gaussian Mixture Model

Table 8 summarizes multimodal fusion methods that integrate visual, physiological and environmental data using both classical and machine learning frameworks. Multimodal fusion methods combine complementary information sources to improve robustness and detection accuracy.

**Table 8:** Multimodal fusion methods.

Method	Description	References
Feature-level fusion + RBF-SVM	Eye features fused with drowsiness indicators and classified using RBF-SVM.	[30]
Physiology + traffic + environment fusion	Multi-factor fusion using RF, SVM, and neural networks; also includes YOLOv3 and CNN weather modules.	[12]
Deep multi-branch biosignal fusion	EEG, EDA, and ECG fused through deep CNN branches.	[26]
Radar fusion CNN-LSTM	FMCW plus TOF radar fusion using attention CNN and LSTM.	[39]
Hybrid RF / LSTM / CNN+Attention fusion	Mixed multimodal architecture for trucking driver monitoring.	[54]
Blink + posture + thermal-comfort multimodal model	SVM-based detection with RBF-SVR prediction using camera, seat, thermal, and GPS inputs.	[46]
Attention-based multimodal deep learning	Multimodal deep learning for connected or automated vehicle prediction with attention components.	[55]

*RF- Random Forest, FMCW- Frequency-Modulated Continuous Wave, TOF- Time of Flight, SVR- Support Vector Regression, GPS- Global Positioning System.

Table 9 shows statistical and behavioral modeling methods used to analyze driving behavior, workload and attention. The table highlights their importance in validating experimental findings. These approaches give interpretive insight into human factors and cognitive processes.

**Table 9:** Statistical and behavioral modeling methods.

Method	Description	References
Repeated-measures ANOVA / rmANOVA	Within-subject behavioral or workload comparison.	[11,13,16,17,19,56]
ANOVA combined with clustering	Clustering used first, then ANOVA for behavioral differences.	[51]
GLMM	Generalized linear mixed modeling for physiological or behavioral effects.	[42]
GEE	Generalized Estimating Equations for repeated driving observations.	[57]
Logistic regression	Used for sleepiness, emotion, or profile-based prediction.	[21,58,59]
Mediation / PROCESS analysis	Mediation or moderated-effects modeling in behavior or attention studies.	[6,60]
Polynomial / surface / ordinal / logistic regression	Higher-order behavioral response modeling.	[17,61]
Entropy measures	Sample-entropy-based behavioral or visual complexity analysis.	[3]
SAGAT-based situation-awareness analysis	Situation-awareness scoring without machine-learning classification.	[62]
GLM + SPM12 voxel modeling	Neurobehavioral analysis using GLM in imaging contexts.	[63]
t-tests / hypothesis testing	Paired or independent tests used without machine-learning classifiers.	[31,64,65]

* ANOVA- Analysis of Variance, rmANOVA- Repeated-measures Analysis of Variance, GLMM- Generalized Linear Mixed Model, GEE- Generalized Estimating Equations, SAGAT- Situation Awareness Global Assessment Technique, GLM- General Linear Model, SPM12- Statistical Parametric Mapping-12,

Table 10 summarizes psychometric and questionnaire-based approaches used in driver-state research. These methods focus on subjective assessments of behavior, emotion and perception through structured surveys and latent-variable modeling. The table demonstrates their role in capturing psychological constructs that are difficult to measure through sensors.

**Table 10:** Psychometric/non-algorithmic methods.

Method	Description	References
EFA / CFA / SEM	Questionnaire-based latent-variable modeling.	[27]
SEM	Structural equation modeling in psychometric or crash-proneness studies.	[60,66]
CBSEM	Covariance-based SEM in survey-driven behavioral studies.	[67]
Correlation + multiple linear regression	Psychometric association modeling without sensing.	[5,7]
Logistic regression on psychometric profiles	Survey-based behavioral prediction.	[58]
Factor analysis / SEM-type survey modeling	Acceptance- or questionnaire-focused modeling.	[68]
Questionnaire-only psychometric profiling	Trait, emotion-regulation, or aggression-scale modeling without algorithmic sensing.	[69]

*EFA- Exploratory Factor Analysis, CFA- Confirmatory Factor Analysis, SEM- Structural Equation Modeling, CBSEM – Covariance-based Structural Equation Modeling

## 4 Discussion

### 4.1 Vision-based sensing: High adoption, variable reliability

Vision-based approaches were the most adopted sensing modality across the reviewed literature (n = 22 studies). Their reported performance however varied across study settings and datasets. Studies using RGB cameras achieved high headline accuracies. A CNN-based YOLOX and RetinaFace system for distraction detection reported object detection accuracy averaging 97.1% [33], a 3D-CNN-LSTM architecture for drowsiness detection achieved 95.3% [35], and an Inception-ResNet-based face recognition classifier reported 98.4–99.2% on standard benchmarks [36]. Attention-based networks such as AG-Net reached 99.70% on the AUC-V1 distracted-driver dataset and 96.65% on AUC-V2 [38]. However, these high figures frequently fail to transfer to naturalistic conditions. Studies using near-infrared cameras demonstrated the sensitivity to environmental factors directly: a user-specific RBF-SVM drowsiness detector operating with NIR video reported no numeric accuracy, citing performance variability across lighting and occlusion conditions [30]. A gaze-zone estimation system using IR cameras reported accuracies ranging from 73.1% to 83.1% depending on time of day and whether the driver wore glasses [32]. Thermal imaging combined with rule-based landmark detection produced no reportable accuracy under real-world conditions [70]. Studies relying on RGB cameras for naturalistic gaze tracking similarly reported non-reportable performance when applied outside controlled settings [71,72]. Face-based emotion recognition using a customized VGG-19 architecture achieved only 66% on the FER2013 and related datasets. This underscores the difficulty of generalizing from controlled image corpora to in-vehicle conditions [28]. The pattern across driver-state detection contexts – distraction [31,38], drowsiness [30,32,35,59,70], emotion [28,36], and gaze [71,73] – is consistent. The average reported accuracies exceeded 90% in several of these studies. Performance however depreciates under changes in illumination, head orientation, and facial occlusion, conditions that are routine in naturalistic driving. This pattern recurs across studies using RGB, near-infrared, and thermal cameras. This suggests that the problem is inherent to the sensing principle rather than to specific hardware choices.

### 4.2 Physiological sensing: Signal consistency at the cost of practicality

Physiological sensing – primarily EEG, ECG, EOG, and EDA – produced consistent signal outputs across the studies in which it was employed. A stacked autoencoder and attention-LSTM system applied to EEG, facial EMG, pulse rate, respiration, and GSR data achieved 98.63% accuracy in a simulator study [4], and a multi-branch deep CNN fusion of EEG, EDA, and ECG signals achieved 82% under leave-one-subject-out cross-validation [26]. LSTM applied to three-layer EEG signals reached 85.6% in a train-driving context [24]. Ensemble classifiers (hard-voting across Decision Tree, RF, Gradient Boosting, LightGBM, CatBoost, AdaBoost) on ECG-derived features achieved 64–74% in a VR driving simulator [21]. For wrist-worn sensors, a classical ML comparison of KNN, SVM, RF, XGBoost, and LightGBM on PPG and EDA data reached 88.7% binary and 85.6% three-class accuracy [43]. Drowsiness detection from ECG, HR, and HRV using logistic regression on a closed-loop driving track reported AUC of 78–100% in younger drivers and 86–94% in older drivers [59]. Thermal-plus-seat-pressure sensing combined with RBF-SVR for drowsiness prediction achieved 83% in real-highway conditions [46]. Studies on mental workload using physiological signals and classical ML reported metric coverage across accuracy and sensitivity [11,12,65,74]. EEG-based studies however, focused on behavioral responses in emergency driving tasks reported no classification accuracy but provided validated physiological markers through ANOVA and GLMM [22]. Review-level contributions synthesized the physiological sensing evidence base across ECG and HRV for fatigue [25] and the broader landscape of stress, road conditions, and driving environment [75]. A recurring limitation across many physiological studies was equipment intrusiveness: wearable electrode arrays and monitoring devices constrained driver movement and are unlikely to be acceptable in consumer-facing Level 3 systems without significant miniaturization advances [56].

### 4.3 Vehicle-performance sensing: Structured data, limited representation

The use of steering entropy, SDLP, and pedal pressure as proxies for driver attentiveness was underrepresented in the reviewed literature despite offering a non-intrusive, equipment-free alternative to physiological and vision-based approaches. A generalized Pareto distribution applied to simulator-derived takeover-time data for takeover-threshold detection achieved a classification accuracy of 90.38% for older drivers [2], while an LightGBM classifier on pre-lane-change driving features reached 92.3% [49]. In contrast, the majority of vehicle-performance studies did not report classification metrics. Repeated-measures ANOVA on steering angle and lateral position data yielded validated behavioral indicators of distraction without a classifier output [3]. Simulator-plus-eye-tracker studies of lane-changing behavior, visual workload and situational awareness in freeway exit areas similarly reported no accuracy values [57,61,62,76]. Studies on tunnel driving behavior used CAN-bus, Mobileye, camera, and GPS combined with K-means clustering and ANOVA to identify lane-deviation patterns. These however didn’t produce a classifier accuracy figure [51]. Additional vehicle-performance contributions examined takeover response timing and driver workload in conditionally automated scenarios [42,77,78], drowsiness indicators through naturalistic driving data [48,54], and intersection risk warning performance [79]. The structured, numerical nature of vehicular data proved well-suited to classical ML classifiers. The primary limitation identified across these studies was ecological validity: the behavioral cues produced are highly dependent on the specific simulator design and takeover event configuration [63]. This limits the generalizability of findings across experimental settings.

### 4.4 Multimodal fusion: Performance benefits and implementation trade-offs

Multimodal fusion approaches – combining two or more of the above sensing modalities – consistently produced balanced performance profiles across all reported metrics. Compared with single-modality methods, several fusion studies reported a broader range of performance metrics. This gave a more balanced evaluation profile. A multi-sensor fusion system combining galvanic skin response, driving simulator outputs, and webcam data for emotion classification achieved 92.0–92.9% accuracy [65]. A real-world expressway study combining ECG, EDA, video (front and rear), and RF/SVM/NN classification with CNN-based vehicle detection reached 97.8% accuracy [12]. A deep CNN fusion of EEG, EDA, and ECG in a driving simulator context achieved 82% under cross-subject validation [26]. Studies combining physiological signals with simulator-based behavioral measures of takeover performance found that emotional instability – assessed via joint physiological and vehicle-performance indicators predicted takeover quality [53]. Train-driving attention monitoring using multi-biosignal fusion with LSTM achieved 85.6% [24]. Driver mental engagement studies applying deep CNN fusion to EEG, EMG, and EDA in simulator conditions demonstrated the advantage of combining physiological modalities over single-signal approaches [26,65]. Other fusion configurations incorporating CAN-bus, camera, GPS, Mobileye, and inertial guidance system data identified driving pattern deviations in urban tunnels [51] and characterized drowsiness progression under real-highway thermal comfort conditions [46]. Conceptual frameworks specifically addressing multimodal cooperation between driver and automation in Level 3 systems provided methodological guidance for fusion architecture design. Utilizing multimodal fusion techniques covers up the bottlenecks of individual modalities. This yields a more robust representation of driver state [64]. Despite the ability to compensate for the limitations of individual sensing modalities, higher performances recorded were achieved under controlled experimental conditions. These depend on accurate sensor synchronization, stable signal quality, reliable missing-data handling and increased computational resources. Therefore, multimodal fusion should not just be judged by classification accuracy or metric coverage, but also by its latency, implementation complexity, robustness to real-world driving conditions and feasibility for continuous in-vehicle monitoring.

### 4.5 Data-flow architecture and real-time constraints in driver state detection

The studies reviewed provide extensive coverage of sensing modalities and how they are classified. However, less attention is given to data-flow architecture that govern the conversion of sensor inputs into driver-state outputs. Driver-state detection systems are not defined just by their sensing modality or classification algorithm; but by the sequence of processing stages through which data is transformed. These stages are signal acquisition, pre-processing, feature extraction, model inference and decision output. Across the reviewed literature, the degree of processing required between input signals and classification output were studied. From that, some dominant data flow architecture were identified.

#### 4.5.1 Direct behavioral detection pipelines.

Vision-based systems used relatively short data flow pipelines. Raw image or video input is processed directly through object detection or classification networks. CNN-based architecture are used to extract facial features and classify driver states such as drowsiness and distraction from image data [28,33]. Attention-based and spatiotemporal CNN models also process video streams to detect yawning and gaze direction [35,38].

In the above systems, the pipeline typically consists of image acquisition and feature extraction through direct classification and convolutional layers. The absence of extensive intermediate processing stages enables relatively low latency inference. These would be suitable for real-time applications. Despite their low latency, their performance hinges on environmental conditions such as illumination, occlusion and head orientation, as discussed in Section 4.1

#### 4.5.2 Feature engineering pipelines for physiological signals.

Physiological sensing approaches exhibit more complex data flow structures. Raw biosignals such as ECG, EEG and EDA require pre-processing steps which include noise filtering, signal segmentation and artefact removal before meaningful features can be extracted. Heart Rate Variability (HRV) features derived from ECG signals are commonly used as inputs to classical machine learning classifiers such as SVM, Random Forest and ensemble models [21,43]. EEG-based systems also require spectral decomposition prior to classification using LSTM and other deep learning architecture [24].

This structured approach needed in making physiological signals useful improves interpretability and signal consistency. However, it introduces processing latency, increasing the potential for cumulative error across stages. Due to this, many physiological systems are not utilised in real-time deployment scenarios.

#### 4.5.3 End-to-end deep learning pipelines.

A subset of studies employs end-to-end deep learning architecture. They aim to minimise manual feature engineering by learning representations directly from raw data. Stacked autoencoder, CNN architecture and attention-based LSTM models [4,26] integrate feature extraction and classification within a single model. This reduces the need for explicit intermediate processing stages.

Although end-to-end models can capture temporal and nonlinear relationships within data, they require large datasets and significant computational resources. This hinders real-time adaptation.

#### 4.5.4 Multimodal fusion pipelines and synchronisation constraints.

Multimodal systems had the most complex data flow architecture identified. These systems combine inputs from multiple sensing modalities to improve robustness and detection accuracy. Fusion frameworks integrating ECG, EDA, video and environmental data have been implemented using combinations of Random Forest, SVM and neural networks [12]. Deep multi-branch CNN architecture have also been used to fuse EEG, EDA and ECG signals [26].

In these systems, the data flow pipeline includes parallel pre-processing and feature extraction for each modality. This is followed by feature-level and final classification. A challenge with such architecture is the temporal synchronisation of heterogeneous data streams. Differences in sampling rates, signal latency and data quality can introduce misalignment. This affects classification performance and system stability.

#### 4.5.5 Real-time versus offline detection constraints.

Another limitation identified across the reviewed literature was the lack of explicit differentiation between offline and real-time detection systems. Many studies report high classification accuracy without specifying whether the system operates under real-time constraints. Vision-based systems are more readily adapted for real-time deployment due to their relatively direct processing pipelines. In contrast, physiological and multimodal systems often rely on multi-stage processing pipelines that introduce latency, making real-time deployment challenging.

This distinction is particularly important for SAE Level 3 takeover monitoring because the system must determine driver readiness before or during the limited takeover window, rather than only classify driver state after data have been collected. Previous studies have examined takeover request times of approximately 3−12 s, while mean driver takeover times of about 3.25–3.63 s have been reported under different driver conditions [2]. These relatively short intervals place additional importance on minimizing sensing, preprocessing and inference latency. Methods based on camera-derived gaze, eyelid closure, head pose, facial orientation and vehicle-control signals, however, are more suitable for real-time monitoring because they can be collected continuously and processed with relatively low latency. However, their reliability may decrease under illumination changes, occlusion, glasses use, head rotation and scenario-specific driving conditions. Physiological methods such as EEG, ECG, EOG, EDA, HRV and respiration provide richer information about internal states such as fatigue, vigilance, stress and workload; their use in production Level 3 vehicles is constrained by sensor intrusiveness, calibration requirements, signal artefacts, pre-processing demands and latency. Therefore, practical driver-state monitoring requires balancing classification performance with latency, deployability, robustness and user acceptability.

The time-sensitive nature of SAE Level 3 transitions reinforces the need to evaluate data-processing latency. Thus, edge computing allows the translation of driver-state detection models from offline analysis to real-time vehicle deployment. Processing sensor data locally on embedded computing platforms close to the various sensors will help reduce latency and limits dependence on network connectivity. For multimodal systems, edge computing can enable synchronized preprocessing, feature extraction, fusion and decision making within limited time constraints. However, edge computing does not eliminate all latency sources; The number of processing stages in a data flow pipeline directly influences system latency, computational load, and robustness. For Level 3 systems where takeover decisions must be made within a limited time window, these trade-offs are critical.

### 4.6 Inconsistent data and reporting methods

The reviewed studies reveal that there are a number of problems in the reporting and interpretation of the driver-state detection and assessment in conditional automated driving. The inconsistencies in sensor results, inconsistencies in data collection circumstances, and inconsistencies in report methods prevent the comparability of the results across the literature. Several review and conceptual contributions mapped these challenges at the field level. A systematic review of physiological fatigue and drowsiness monitoring [25], a broad review of driver stress across traffic and environmental conditions [75], a cognitive-load and task-switching analysis for semi-autonomous vehicles [10], a review of existing technologies for identifying abnormal driving [80], a naturalistic driving study of awareness and speed behavior [81], and a qualitative study of autopilot as a default cognitive mode [82] collectively document the fragmentation of the evidence base. Non-sensing and psychometric studies contributed evidence on emotional regulation and road aggression [7,69], personality traits and driving distraction [67], mentalization and road aggression [60], fatigue and persistent performance impairment in new drivers [6], driver uncertainty in lane-change decisions [61], situation awareness assessment by experts [83], and automated vehicle acceptance [68]. These were investigated without algorithmic sensing, highlighting that driver state has dimensions that camera and biosignal systems alone do not capture. Ambient conditions such as the change of the illumination and the change of the head orientation affected vision-based sensors. Such factors caused the instability in the gathered facial or postural cues, which led to drastic changes in sensitivity and F1-scores. Vision-based systems, even though widely used in the studies under analysis, did not allow generalizing their performance to various tasks, devices, or scenarios on the road, due to the variety of operational environments.

The existence of single and multi-metric studies (Table 11) led to the absence of balance in the interpretation of performance, which made it difficult to determine uniform results. These discrepancies could be seen through the dispersion of measures on the 85 trials analyzed. This inconsistency also varied across sensing modalities, with substantial differences in the reporting frequencies of accuracy, sensitivity, F1-score, AUC, and reaction time (Fig 4).

**Table 11:** Summary of evaluation metrics reported across the included studies.

Metric	Number of Studies Reporting (n)	Percentage (%) of Total Studies (N = 85)	Analytical Implication
Overall Classification Accuracy	38	44.7%	Most common metric; indicates surface-level validation tendency
Reaction-Time Impacts	61	71.8%	Reflects behavioral emphasis and human-factor assessment
Sensitivity / Recall	24	28.2%	Moderately reported; limited by missing confusion matrices
F1-Score	19	22.4%	Underrepresented; essential for imbalanced datasets
AUC	9	10.6%	Least reported; advanced diagnostic measure rarely included

The inconsistent reporting of evaluation metrics limits cross-study interpretation. This inconsistency matters because driver-state detection is often concerned with identifying rare but safety-critical states rather than simply maximizing overall correct classification. Cross-study numerical comparisons should therefore be interpreted cautiously, since reported values of accuracy, F1-score, AUC, or sensitivity are influenced by differences in driver-state labels, class distributions, sampling strategies, sensing modalities, experimental tasks, simulator fidelity, environmental conditions, and validation procedures. A high accuracy value obtained from a controlled, balanced dataset may not indicate the same practical reliability as a lower value obtained under more naturalistic or operationally complex conditions.

### 4.7 Inconsistent definition of driver-state constructs

A further challenge was inconsistency in both the definition and operationalization of driver-state constructs. Constructs such as distraction were investigated using different operational measures, including behavioral indicators, physiological markers, secondary-task performance, and simulator-based task manipulations. These approaches are not necessarily contradictory, as they may provide complementary measures of the same underlying construct; however, differences in thresholds, temporal windows, task designs, and ground-truth criteria can lead to substantial variation in labelling and evaluation across studies.

Studies that used 2x2 classification tables proved to be very sensitive to variations in the true positives, false positives, false negatives, and true negatives. Any changes in the balance of these classes resulted in significant changes in sensitivity, specificity and F1-scores, although the underlying performance of the classifier was the same.

### 4.8 Heterogeneity in methodology used

**Fig 6:**
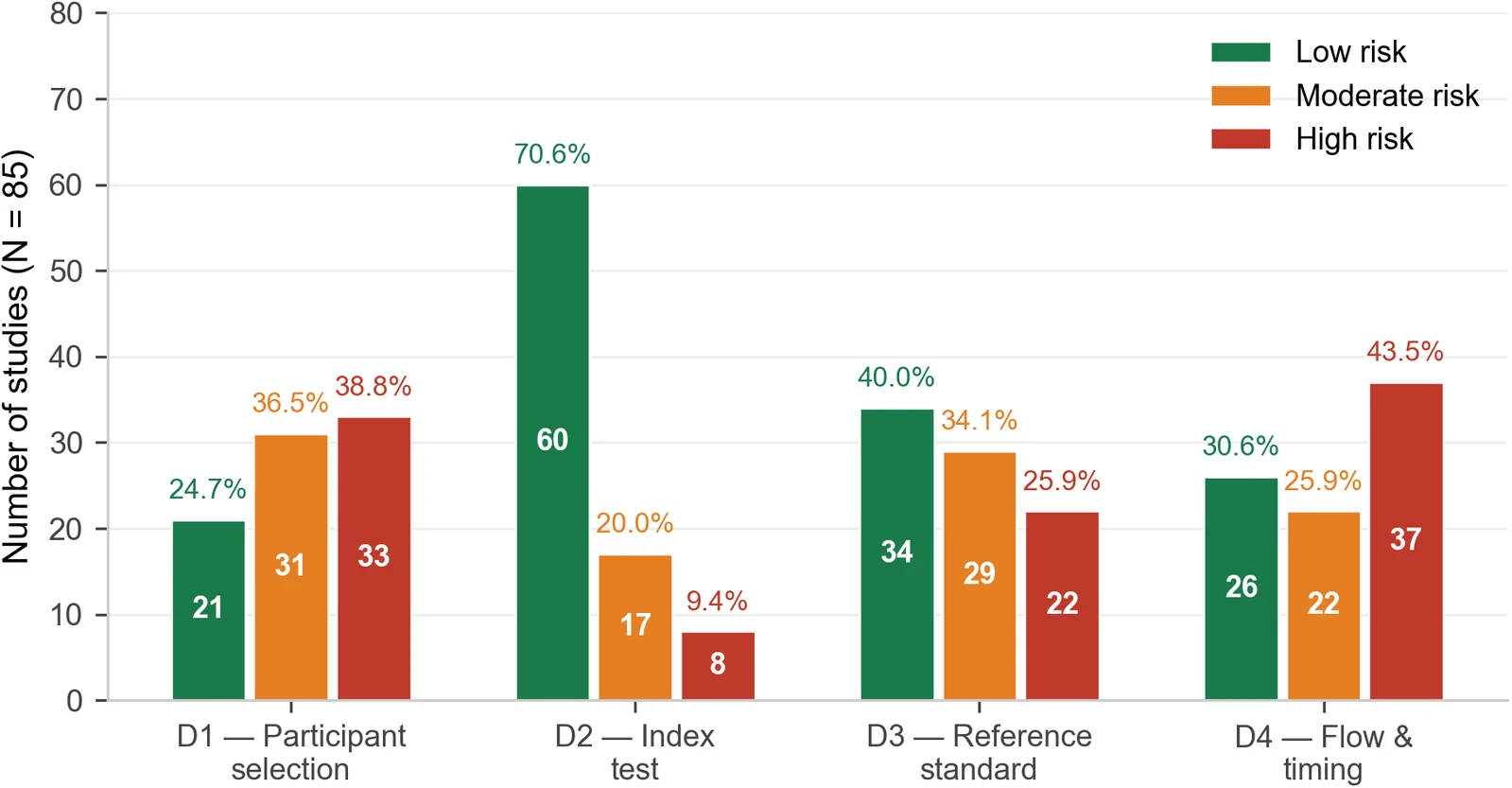
Risk-of-bias assessment across four RoBVIS domains.

Much of the challenge regarding methodological consistency was also revealed in RoBVIS evaluation (Table 12 & Fig 6). In seventy percent of the studies, low-risk was established in the index-test domain but high-risk assessments were located mostly in the areas of participant-selection and flow-and-timing. The seventy high-risk instances between the two domains showed that many of the studies used non-representative samples, lapsed time between sensor data and that they failed to synchronise behavioral, visual, and physiological sensor data. The domain of reference standard had twenty-two high-risk ratings which can be explained by the reliance on subjective ground-truth definitions as opposed to objective physical standards. Such issues reduced the confidence in reported performance claims. They also revealed major methodological gaps across many studies.

**Table 12:** Summary of RoBVIS ratings across the included studies.

Domain	Low Risk (n)	Moderate Risk (n)	High Risk (n)	Dominant Source of Bias
Participant Selection	21 studies (24.7%)	31 studies (36.5%)	33 studies (38.8%)	Convenience sampling, limited demographic diversity
Index Test	60 studies (70.6%)	17 studies (20.0%)	8 studies (9.4%)	Incomplete validation details or absent cross-testing
Reference Standard	34 studies (40.0%)	29 studies (34.1%)	22 studies (25.9%)	Subjective or inconsistent ground-truth definitions
Flow & Timing	26 studies (30.6%)	22 studies (25.9%)	37 studies (43.5%)	Unclear sensor synchronization or time-lag issues

### 4.9 Limitations of the evidence and review process

Many of the reviewed studies used simplified environment or constant vigilance procedures, which do not sufficiently assess the complexity of real-time changes in Level 3 driving. Though these environments helped in the precise measurement of physiological or behavioral responses, the controlled environment also restricted the use of the results to real-life scenarios. Such experimental conditions often did not capture environmental unpredictability [84,85], multitasking demands, effects of motion and the real-time system requirements characteristic of real-world Level 3 driving.

Furthermore, the recognition of driver-state was based on the other factors such as the cognitive load, the visual load and the emotional state. These determinants required measurement to be consistent across research, but they were defined differently. There was a varied influence of emotional factors on driving behavior in various research environments, such as anger, annoyance, and stress. The variability in these determinants led to the dissimilarity in the operationalisation of driver state.

Other problems arose in the time aspects of the acquisition performance. Reaction-time effects were measured with different methodologies; some of them used gaze shifts, some used data of pedal-pressure or button clicks. The differences made the comparison hard and complicated the meaning of a driver who is distracted, fatigued or emotionally disturbed to react to a takeover request.

Problems connected to sensor stability, changes in data sets, differences in metric reporting and bias patterns, divergent definitions, and environmental limitations make finding reliability and generalizability to real-world problems in driver-state detection methods challenging.

Methodological limitations exist for the review process utilised: First, the search was limited to just 4 databases. Although these databases cover a substantial proportion of engineering, transportation, computer science and human factors literature, relevant studies indexed outside these sources may have been missed. Also, because the included studies varied across several domains, a quantitative meta-analysis was not conducted. The findings should therefore be interpreted as a narrative and thematic synthesis.

## 5 Future directions and research gaps

The synthesis of 85 studies reveals five specific research gaps that represent the highest-priority needs for advancing driver-state detection in Level 3 automated driving:

### 5.1 Gap 1: Absence of a Level 3-specific benchmark dataset

No publicly available, standardised dataset exists that combines synchronized multimodal data collected during realistic SAE Level 3 takeover events. The reviewed studies used a wide range of proprietary, simulator-based, and repurposed datasets (e.g., FER2013, LISA V2) that were not designed for this context. This gap makes cross-study comparison unreliable and prevents the development of generalizable models. Future research should prioritise the creation and open publication of a Level 3 driver-state benchmark dataset, collected from demographically stratified participants in both simulator and instrumented real-vehicle environments, with standardised TOR event designs and synchronized multi-sensor logging.

### 5.2 Gap 2: Inconsistent definition and operationalization of driver-state constructs

The constructs of ‘distraction,’ ‘fatigue,’ ‘drowsiness,’ and ‘cognitive load’ were operationalised inconsistently across the reviewed literature, making nominal comparisons between studies substantively misleading. No community-agreed operational definitions currently exist for these constructs in the Level 3 takeover context. Future work should establish a taxonomy of driver-state constructs with agreed measurement operationalisations, ideally anchored to objective physiological or behavioral thresholds rather than subjective ratings.

### 5.3 Gap 3: Underrepresentation of diverse driver populations

The RoBVIS assessment identified convenience sampling as the dominant source of bias in Participant Selection (38.8% high-risk). The majority of reviewed studies recruited young, university-educated participants, systematically excluding older drivers, drivers with disabilities, and commercial drivers. Given that Level 3 vehicles will be operated across the full age spectrum and in diverse driving environments, future studies must recruit demographically representative samples and report participant characteristics (age, gender, driving experience, health status) in sufficient detail to enable subgroup analyses.

### 5.4 Gap 4: Insufficient real-world and longitudinal validation

Only 12 out of the 85 reviewed studies were conducted in direct Level 3 automation settings; the remainder used simulators or proxy tasks. Simulator validation is a necessary but insufficient step for systems intended for real-world deployment. Future research must include on-road validation studies with instrumented Level 3 vehicles, conducted under naturalistic conditions including night driving, adverse weather, and high-traffic environments. Longitudinal studies assessing driver-state detection system performance over extended periods (weeks to months) are also absent from the literature and are needed to evaluate reliability under long-term use conditions.

### 5.5 Gap 5: Absence of minimum reporting standards for performance evaluation

The finding that 44.7% of studies reported a numeric accuracy value, while only 10.6% reported AUC (Fig 5), reflects the absence of community-agreed reporting standards. This prevents safety validation, since accuracy-only reporting cannot detect the class-imbalance failure modes most relevant to real-world driver monitoring. The field needs a minimum reporting checklist for driver-state detection studies. At minimum, this checklist should require: classification accuracy, F1-score, sensitivity, AUC or specificity, sample size and class balance, and dataset origin (public or proprietary). A proposed checklist is provided in the supplementary material.

### 5.6 Policy and practice implications

UN Regulation No. 157 [86] requires vehicles equipped with Automated Lane Keeping System to incorporate a driver-availability recognition system capable of detecting whether the driver is present and available to take over the driving task. The regulation also calls for real-world assessment by engineers to supplement the manufacturer’s technical documentation. The European Union, Regulation 2019/2144 [87] further mandates driver drowsiness and attention warning systems: this emphasizes the regulatory importance of reliable driver-state monitoring. Reliable regulatory deployment depends on clearly defined driver states, validation under real-world conditions, robust sensing under degraded or variable conditions and evidence that driver-state monitoring can operate accurately within real-time transition constraints. This need for clearer and more harmonised requirements is consistent with broader fatigue-management evidence showing that inconsistent enforcement and regulatory interpretation can weaken safety practice in transport settings [88]. However, these legal instruments focus primarily on functional requirements, validation procedures, and privacy-related constraints, rather than prescribing a standardized academic reporting set based on metrics such as sensitivity, F1-score, or AUC.

## 6 Conclusion

This review evaluated recent advances in driver-state detection and recognition in conditional automated driving, drawing on studies published mainly between 2021 and 2025. The reviewed studies showed that significant progress has been made across sensing modalities and modeling approaches. However, the field remains constrained by limitations in reliability, comparability and real-world applicability.

A comparative synthesis of the bibliography reveals modality-specific tradeoffs. Vision-based systems, although widely used and capable of achieving high classification accuracies under controlled conditions, show performance degradation under real-world variations in illumination, occlusion and head movement. Physiological sensing approaches demonstrate more stable and internally consistent indicators of driver state. They are also limited by their intrusive nature and reduced practicality when deployed on a large scale. Vehicle-performance-based methods are non-intrusive, but are underutilised and lack robust classification frameworks. In contrast, multimodal fusion approaches consistently demonstrate more balanced and reliable performance. They however introduce additional complexity in synchronisation, computation and system integration.

Despite these advances, the field is characterized by significant fragmentation. A major limitation that was identified across the reviewed studies is the lack of a unified definition of driver state, with behavioral, physiological and subjective criteria applied inconsistently. This inconsistency extends to data collection environments, experimental protocols and performance reporting practices and results in limited comparability across studies. The imbalance in reported evaluation metrics, particularly the dominance of accuracy over more informative measures such as F1-score and AUC, further constrains the interpretation of model performance. In addition, methodological assessment using RoBVIS highlights recurring risks of bias, particularly in participant selection, reference standard definition, and temporal synchronization of multimodal datasets.

Research in this field shows a shift from isolated, single-modality approaches to more integrated ones. Despite the growing shift, many studies still depend on controlled experimental environments that are unable to capture the complexity and unpredictability of real-world Level 3 driving.

Future studies must shift from just incremental improvements in model accuracy to a more structured and standardised research framework. This includes the development of unified definitions and taxonomies for driver state and the adoption of consistent and multi-dimensional evaluation metrics, together with more real-world studies. Only through improved standardisation, methodological rigour and real-world validation can driver-state detection systems achieve the required safety needed in Level 3 automated driving.

## Supporting information

S1 Table Database-specific search strategies used in the systematic review. Search fields, search strings, filters applied, and records identified across ScienceDirect, Web of Science, SpringerLink, and IEEE Xplore.
(DOCX)

S2 Table Proposed minimum reporting checklist for driver-state detection studies. Recommended reporting items covering study context, driver-state operationalization, participant and dataset characteristics, sensing configuration, validation strategy, performance metrics, real-time feasibility, and robustness.
(DOCX)

S1 Data Extracted study data and risk-of-bias assessments used in the systematic review. Workbook containing study-level classification data, study categorization, RoBVIS assessments, and summary risk-of-bias data.
(XLSX)

S1 Checklist PRISMA 2020 for abstracts checklist. Completed checklist indicating whether the applicable PRISMA for Abstracts reporting items are addressed.
(DOCX)

S2 Checklist PRISMA 2020 checklist. Completed PRISMA 2020 checklist indicating where each applicable reporting item is addressed in the manuscript.
(DOCX)
