# Supplementary material for: Driver state detection and recognition in conditional automated driving: A review
Source: PLoS One. 2026 Sep 17;21(9):e0358700. doi: 10.1371/journal.pone.0358700 (PMC13585244; doi:10.1371/journal.pone.0358700)
Supplement: Supplementary file 1 [file pone.0358700.s001.docx]

| **Database** | **Search field** | **Search strategy** | **Filters applied** | **Records identified** |
| --- | --- | --- | --- | --- |
| **ScienceDirect** | Advanced Search (Title, Abstract, Keywords) | ("driver state" OR "driver condition" OR "driver status" OR distraction OR drowsiness) AND ("Level 3" OR "SAE Level 3" OR "conditional automation") AND (recognition OR detection OR classification) | Publication years: 2021-2025; Article type: Research Articles and Review Articles; Subject area: Engineering; Language: English | 191 |
| **Web of Science** | Topic (TS: Title, Abstract, Author Keywords, Keywords Plus) | TS = ("driver state" OR "driver condition" OR "driver status" OR distraction OR drowsiness) AND TS = ("Level 3" OR "SAE Level 3" OR "conditional automation") AND TS=(recognition OR detection OR classification) | Timespan: 2021-2025; Document types: Article and Review Article; Research area: Engineering; Language: English | 92 |
| **SpringerLink** | Advanced Search (Title, Abstract, Keywords) | ("driver state" OR "driver condition" OR "driver status" OR distraction OR drowsiness) AND ("Level 3" OR "SAE Level 3" OR "conditional automation") AND (recognition OR detection OR classification) | Publication years: 2021-2025; Content type: Article; Discipline: Engineering; Language: English | 165 |
| **IEEE Xplore** | Advanced Search (Metadata: Title, Abstract, Index Terms) | ("driver state" OR "driver condition" OR "driver status" OR distraction OR drowsiness) AND ("Level 3" OR "SAE Level 3" OR "conditional automation") AND (recognition OR detection OR classification) | Publication years: 2021-2025; Content type: Journals; Language: English | 11 |

**Total records identified:** 459

- ScienceDirect: 191
- Web of Science: 92
- SpringerLink: 165
- IEEE Xplore: 11

**Additional search processing:** Retrieved records were exported and consolidated in EndNote 21. Duplicate records were removed before title and abstract screening. The complete screening process is presented in the PRISMA flow diagram (Fig 1).
