# Supplementary material for: Driver state detection and recognition in conditional automated driving: A review
Source: PLoS One. 2026 Sep 17;21(9):e0358700. doi: 10.1371/journal.pone.0358700 (PMC13585244; doi:10.1371/journal.pone.0358700)
Supplement: Supplementary file 2 [file pone.0358700.s002.docx]

| **Reporting category** | **Minimum information to report** | **Purpose** |
| --- | --- | --- |
| Study context | Automation level, simulator or real-road setting, driving task, and takeover scenario where applicable | Establishes relevance to real-world SAE Level 3 operation |
| Target driver state | State investigated and its operational definition, such as distraction, fatigue, drowsiness, workload, vigilance, or takeover preparedness | Reduces ambiguity arising from inconsistent labels |
| Participant characteristics | Sample size, age range, sex or gender distribution where available, driving experience, and inclusion/exclusion criteria | Supports assessment of representativeness and generalizability |
| Class distribution | Number or proportion of samples or events in each class | Reveals class imbalance and helps interpret accuracy |
| Dataset origin | Public or proprietary dataset; laboratory, simulator, naturalistic, or real-road source | Supports reproducibility and assessment of ecological validity |
| Sensing configuration | Sensor modality, placement, sampling rate, acquisition conditions, and calibration requirements | Clarifies intrusiveness, deployment requirements, and data quality |
| Data processing | Preprocessing, segmentation window, feature extraction, missing-data treatment, and fusion strategy | Enables methodological replication |
| Model description | Algorithm, architecture, parameter-selection procedure, and decision thresholds | Supports comparison and reproducibility |
| Validation strategy | Train/test split, cross-validation approach, subject-dependent or subject-independent testing, and external validation where available | Indicates whether performance is likely to generalize |
| Confusion matrix | True positives, true negatives, false positives, and false negatives, or equivalent class-wise values | Allows independent calculation and interpretation of metrics |
| Classification metrics | Accuracy, precision, sensitivity/recall, specificity or false-positive rate, and F1-score; report class-wise or macro-averaged values where appropriate | Prevents reliance on accuracy alone |
| Threshold-independent metric | AUC where probabilistic or threshold-based classification is applicable | Evaluates discrimination across decision thresholds |
| Uncertainty reporting | Confidence intervals, standard deviations, or variability across folds, participants, or repeated trials | Shows the stability of reported performance |
| Reaction-time outcome | Definition of reaction time, event reference point, measurement method, and summary statistics where takeover performance is studied | Connects driver-state monitoring to takeover preparedness |
| Real-time feasibility | Offline or real-time operation, processing-window length, inference latency, hardware, and computational requirements | Supports assessment of practical deployability |
| Failure and robustness analysis | Sensor failures, missing signals, false-alarm rates, environmental limitations, and performance under lighting, occlusion, motion, or individual variation | Indicates likely reliability in real driving conditions |
